# The Role of Chest Physiotherapy in Prevention of Postextubation Atelectasis in Pediatric Patients with Neuromuscular Diseases

**Published:** 2013

**Authors:** Nemat BILAN, Bita POORSHIRI

**Affiliations:** 1 Professor of Pediatric Pulmonology, Paediatric Health Research Center, Department of Pediatrics, Tabriz University of Medical Sciences, Tabriz, Iran; 2 Resident of Pediatrics, Department of Pediatrics, Tabriz University of Medical Sciences, Tabriz, Iran

**Keywords:** Chest physiotherapy, Atelectasis, Postextubation

## Abstract

**Objective:**

There are controversial findings in the literature on the effects of chest physiotherapy on postextubation lung collapse in pediatric age group.

Therefore, we aimed to investigate the efficacy of chest physiotherapy in prevention of postextubation atelectasis in pediatric patients.

**Materials & Methods:**

In a case-control study from March 2007 to March 2011, two groups of patients (35 patients in each group) susceptible to lung collapse were enrolled in the study. The studied patients had neuromuscular diseases such as spinal muscular atrophy, Guillain-Barre syndrome, critical illness polyneuropathy/myopathy, and cerebral palsy. The patients were randomly divided into two groups (case and control); The case group underwent daily chest physiotherapy through vibrator and chest percussion and the control group was under supervision. In the latter group, the underlying disease was treated and the lung collapse was managed, if occurred.

**Results:**

The frequency of atelectasis was lower in the case group who received prophylactic chest physiotherapy compared to the control group (16.6% vs. 40%).

**Conclusion:**

Chest physiotherapy as well as appropriate and regular change of position can considerably reduce the rate of pulmonary collapse in pediatric patients.

## Introduction

Endotracheal intubation and mechanical ventilation can lead to trauma and inflammation of the airways and increase secretions of the lungs and subsequently may contribute to post extubation respiratory complications (1). Neonates are at risk of the obstructing effects of accumulating airway secretions, due to immaturity of the respiratory system (2). The excess of secretions can cause bronchial obstruction and lung collapse as a result of absorption of air beyond the obstruction. The presence of lung collapse may require further supports, such as additional oxygen and occasionally reintubation (in 10-30% of cases) for more mechanical ventilation (3).

Chest physiotherapy, an airway clearance technique, is combined of chest wall percussion and vibrations, positioning of the patient for drainage of mucus, and cough and breathing techniques. The technique is believed to reduce respiratory complications through promoting clearance of secretions and consequently improving ventilation of the lungs (4). Since late 1970s, the use of chest physiotherapy techniques for the prevention of postextubation lung collapse has become a part of routine care (5). Nevertheless, there are controversial findings in the literature regarding the effects of chest physiotherapy on the postextubation lung collapse in pediatric age group (2,5-7). Therefore, the aim of the present study was to investigate the role of chest physiotherapy in prevention of postextubation atelectasis in pediatric patients.

## Materials & Methods

In a case-control study from March 2007 to March 2011, 70 patients susceptible to lung collapse were enrolled in the study. 

The patients who were extubated, were randomly divided in two groups (case and control). Patients in the case group underwent daily chest physiotherapy by vibrator and chest percussion by their trained parents or an experienced staff. The control group was just under supervision. In this group, the underlying disease was treated and the lung collapse was managed, if occurred. 

The patients were matched by age, sex and underlying disorder and there were no significant difference between them (table1and 2).

The patients with accidental or self-extubation were excluded. The patients and their parents were convinced about advantageous of the study and the details of informed consent were clearly explained to them and after this process, they signed it. 

The data were analyzed by chi-square test and p-value<0.05 was considered as significant. This study was approved by the Ethics Committee of the Children Hospital.

## Results

The findings of the study showed that the frequency of atelectasis was lower in the patients of the case group who received prophylactic chest physiotherapy compared to control Group (16.6% vs 40% respectively; p<0.04).

It should be reminded that five patients from the case group and four patients from the control group were excluded because of death or early discharge from hospital.

**Table 1:** Characteristics of Patients and The Main Findings

	**case**	**control**	**p-value**
Number of patients	30 (35)	31(35)	NS*
age	2-12±1	2-12±2	NS
Sex ratio (male/Female)	3/2	4/3	NS
Postextuation atelectasis	5(30)=16.6%	12(30)=40%	p<0.04

* Ns: not significant

**Table2:** Distribution of Diseases

	**case**	**control**	**p-value**
Guillain-Barré syndrome	11	10	NS
Spinal muscular atrophy	11	10	NS
Critical illness neuropathy	6	5	NS
Cerebral palsy	7	10	p<0.04

## Discussion

The present study revealed that atelectasis was less detected in the patients receiving chest physiotherapy compared to the control group. This finding complements that of Finer et al. who investigated the role of chest physiotherapy in the prevention of postextubation atelectasis in neonates (5). In contrast, a few studies targeted at the role of chest physiotherapy in prevention of the postextubation atelectasis, did not support our finding. In a before-and-after study, Bloomfield et al. reported no benefit of postextubation chest physiotherapy in post extubation collapse (7). urthermore, Al-Alaiyan and colleagues stated that postextubation chest physiotherapy did not prevent atelectasis in extubated infants (6). In a study by Bagley et al., no significant difference was found in the rate of postextubation collapse between the chest physiotherapy group and control group (8). In a systematic review, Flenady and Gray (2002) could not give a clear direction for the role of active chest physiotherapy for babies being extubated from mechanical ventilation in neonatal intensive care settings (2). Their conclusion was based on the results of four trials (5,6,9, 10), two of which were carried out several years ago (2). Therefore, applicability of the results of these two studies to current practice might be compromised due to recent advancements in neonatal care (2).

Apart from the role of chest physiotherapy in the prevention of postextubation atelectasis in neonates, the efficacy of this intervention has been assessed in prevention of postoperative atelectasis. Reines and coworkers demonstrated no benefit from the use of chest physiotherapy, routinely in their pediatric postoperative cardiac surgery patients (11). In addition, they warned the clinicians about the possible harmful outcome of application of the chest physiotherapy, that is, increased incidence of atelectasis in this group (11). Contrarily, Cavenaghi et al. reviewed the relevant studies and highlighted the effectiveness of physiotherapy in reduction of the risk and/or treatment of pulmonary complications, such as atelectasis caused by surgical procedure in children with congenital heart disease (12). 

On the other hand, Deakins and Chatburn compared intrapulmonary percussive ventilation with conventional chest physiotherapy to determine their effects on improving atelectasis in pediatric patients. They found that clinically important improvement in atelectasis was more detected in patients who received intrapulmonary percussive ventilation therapy compared to chest physiotherapy (13). 

In conclusion, conventional chest physiotherapy seems to be effective in the prevention of postextubation atelectasis in pediatric patients. It is recommended that further randomized controlled trials be performed with large number of infants in order to evaluate the role of prophylactic chest physiotherapy in the postextubation period in neonates and its adverse effects.

## References

[1] Jorgensen J, Wei JL, Sykes KJ, Klem SA, Weatherly RA, Bruegger DE, Latz AD, Nicklaus PJ (2007). Incidence of and risk factors for airway complications following endotracheal intubation for bronchiolitis. Otolaryngol Head Neck Surg.

[2] Flenady VJ, Gray PH Chest physiotherapy for preventing morbidity in babies being extubated from mechanical ventilation. Cochrane Database Syst Rev.

[3] Odita JC, Kayyali M, Ammari A (1993). Post-extubation atelectasis in ventilated newborn infants. Pediatr Radiol.

[4] Balachandran A, Shivbalan S, Thangavelu S (2005). Chest physiotherapy in pediatric practice. Indian Pediatr.

[5] Finer NN, Moriartey RR, Boyd J, Phillips HJ, Stewart AR, Ulan O (1979). Postextubation atelectasis: a retrospective review and a prospective controlled study. J Pediatr.

[6] Al-Alaiyan S, Dyer D, Khan B (1996). Chest physiotherapy and post-extubation atelectasis in infants. Pediatr Pulmonol..

[7] Bloomfield FH, Teele RL, Voss M, Knight DB, Harding JE (1998). The role of neonatal chest physiotherapy in preventing postentubation atelectasis. J pediatr.

[8] Bagley CE, Gray PH, Tudehope DI, Flenady V, Shearman AD, Lamont A (2005). Routine neonatal postextubation chest physiotherapy: a randomized controlled trial. J Paediatr Child Health.

[9] Vivian-Beresford A, King C, Macauley H (1987). Neonatal post-extubation complications: the preventive role of physiotherapy. Physiother Can.

[10] Bagley C, Flenady V, Tudehope D, Gray P (1999). The role of postextubation chest physiotherapy: A randomized controlled trial.

[11] Reines HD, Sade RM, Bradford BF, Marshall J (1982). Chest physiotherapy fails to prevent postoperative atelectasis in children after cardiac surgery. Ann Surg.

[12] Cavenaghi S, Moura SC, Silva TH, Venturinelli TD, Marino LH, Lamari NM (2009). Importance of pre- and postoperative physiotherapy in pediatric cardiac surgery. Rev Bras Cir Cardiovasc.

[13] Deakins K, Chatburn RL (2002). A comparison of intrapulmonary percussive ventilation and conventional chest physiotherapy for the treatment of atelectasis in the pediatric patient. Respir Care.

